# Isolated atrial infarction complicated by sick sinus syndrome and atrial fibrillation: a case report

**DOI:** 10.1093/ehjcr/ytae692

**Published:** 2024-12-23

**Authors:** Takanori Maeda, Soichiro Yamashita, Koji Kuroda, Masanori Okuda

**Affiliations:** Department of Cardiology, Hyogo Prefectural Awaji Medical Center, Sumoto, Japan; Department of Cardiology, Hyogo Prefectural Awaji Medical Center, Sumoto, Japan; Department of Cardiology, Hyogo Prefectural Awaji Medical Center, Sumoto, Japan; Department of Cardiology, Hyogo Prefectural Awaji Medical Center, Sumoto, Japan

**Keywords:** Isolated atrial infarction, Sinus node artery, Sick sinus syndrome, Atrial fibrillation, Catheter ablation, Case report

## Abstract

**Background:**

Atrial infarction is a complication of myocardial infarction with ventricular infarction; however, isolated atrial infarction (IAI) has rarely been reported. Herein, we report a case of IAI associated with sick sinus syndrome and atrial fibrillation (AF).

**Case summary:**

An 83-year-old woman was brought to the emergency department with a complaint of general malaise. An electrocardiogram at the time of her arrival showed a junctional rhythm with sinus arrest (SA) at a heart rate of 30 bpm; therefore, temporary pacing was placed urgently, and coronary angiography (CAG) was performed. Coronary angiography showed a solitary occlusion of the sinus node (SN) artery originating from the proximal portion of the right coronary artery. Therefore, revascularization was performed for the occluded SN artery to recover SN function. Bradycardia persisted for several days after the procedure but returned to normal sinus rhythm on day 10. However, during hospitalization, AF attacks frequently appeared with an SA for up to 10 s at AF termination, which is known as the bradycardia–tachycardia syndrome. Catheter ablation (CA) was performed for AF, and no recurrence of AF or bradycardia occurred thereafter. She was discharged without any symptoms.

**Discussion:**

We have experienced a patient who underwent revascularization for the occluded SN artery and CA for paroxysmal AF following IAI, which evaded permanent pacemaker implantation.

**Learning points**

- Isolated atrial infarction is poorly recognized and understudied. Occlusion of sinus node artery results in acute-onset sick sinus syndrome. It is difficult to diagnose isolated atrial infarction without coronary angiography. Revascularization of the occluded sinus node artery can recover the sinus node function and consequently can avoid permanent pacemaker implantation.
- This is the rare description of electro-anatomical mapping showing the scar area around sinus node created by atrial infarction. We successfully treated a brady-tachycardia syndrome by control atrial fibrillation by catheter ablation.

## Introduction

Atrial infarction, a common complication of ventricular infarction, is associated with various types of supraventricular arrhythmias, including atrial fibrillation (AF) and sick sinus syndrome (SSS).^1,2^ However, few papers have reported isolated atrial infarction (IAI), and detailed causes and pathologies are still unknown.^3^ Consequently, this condition is difficult to diagnose and may have been overlooked. Herein, we describe a case of IAI complicated by SN dysfunction and AF, which were successfully treated by coronary revascularization and catheter ablation (CA).

## Summary figure

**Figure:**
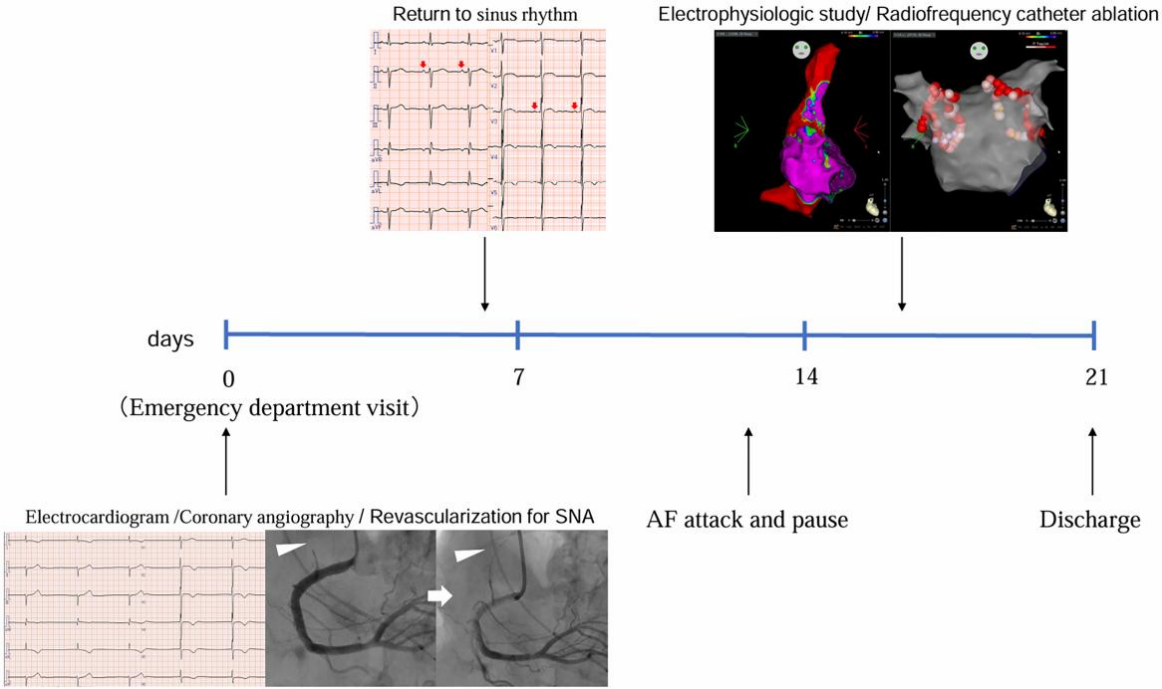


## Case presentation

**Figure 1:**
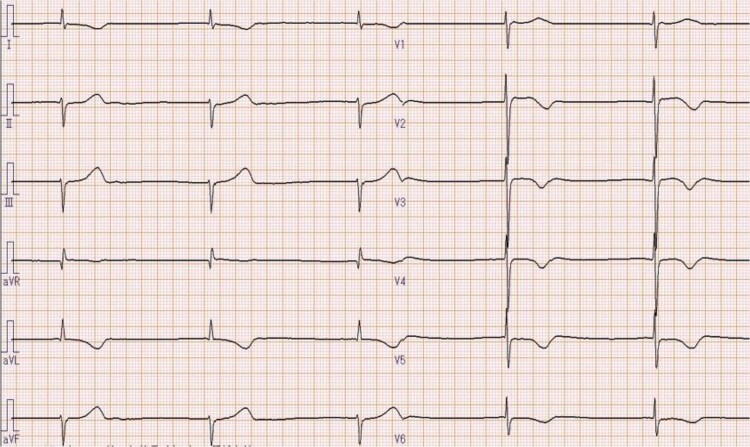
Electrocardiogram in the emergency department showed a junctional rhythm of 32 bpm without P waves.

**Figure 2:**
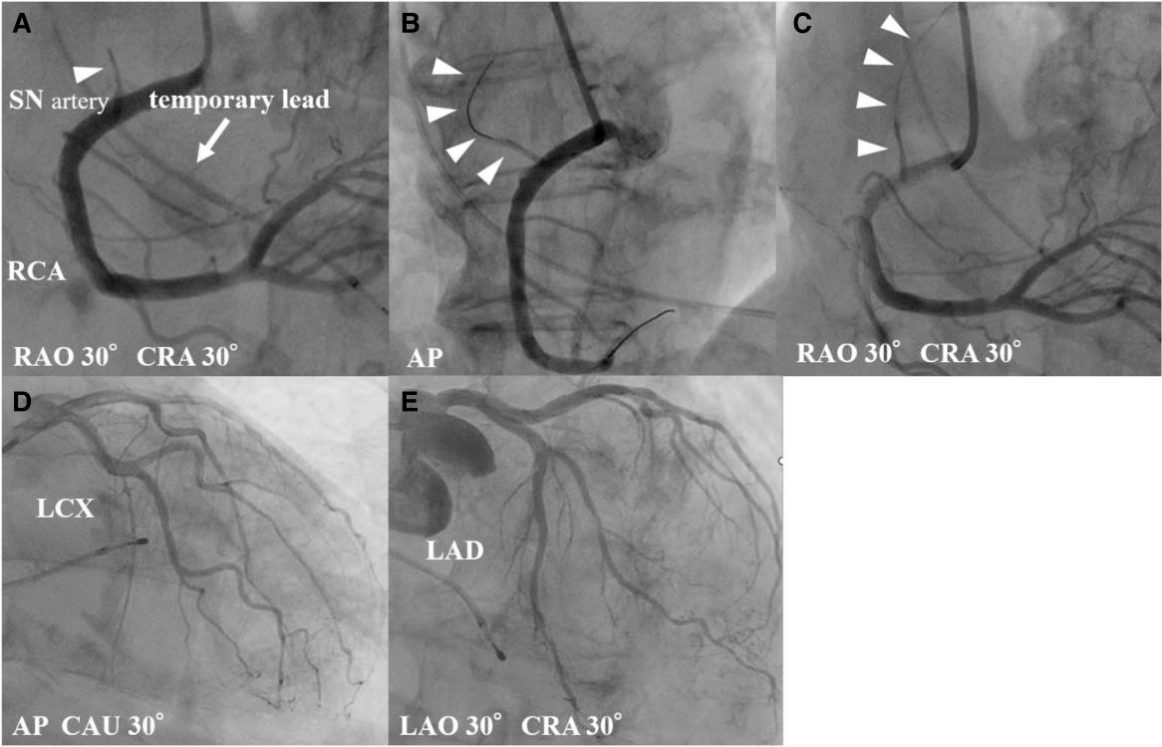
Coronary angiography reveals an isolated occlusion of the sinus node artery branching from the proximal right coronary artery (*A, C, D, E*). A wire successfully crossed the sinus node artery, and the lesion was bougied with a microcatheter (*B*). The final coronary angiography showed good blood flow in the sinus node artery. Coronary angiography revealed no significant stenosis in left coronary artery. LAD, left anterior descending artery; LCX: left circumflex artery.

**Figure 3:**
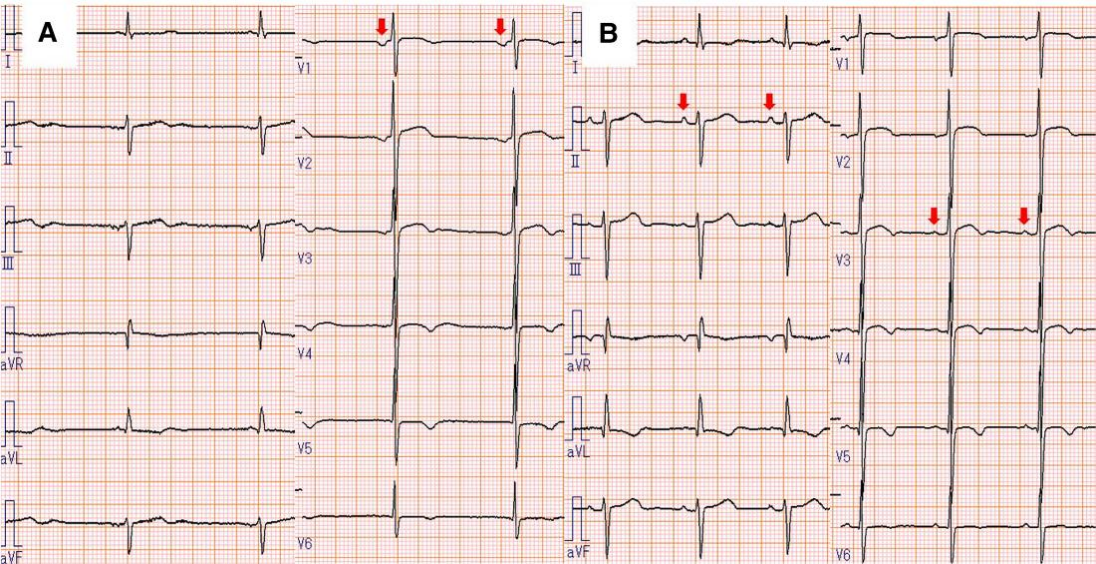
On day 4 after admission, an atrial escape rhythm with negative P waves in V1 started to appear (*A*). Sinus rhythm recovered on day 10 (*B*).

**Figure 4:**
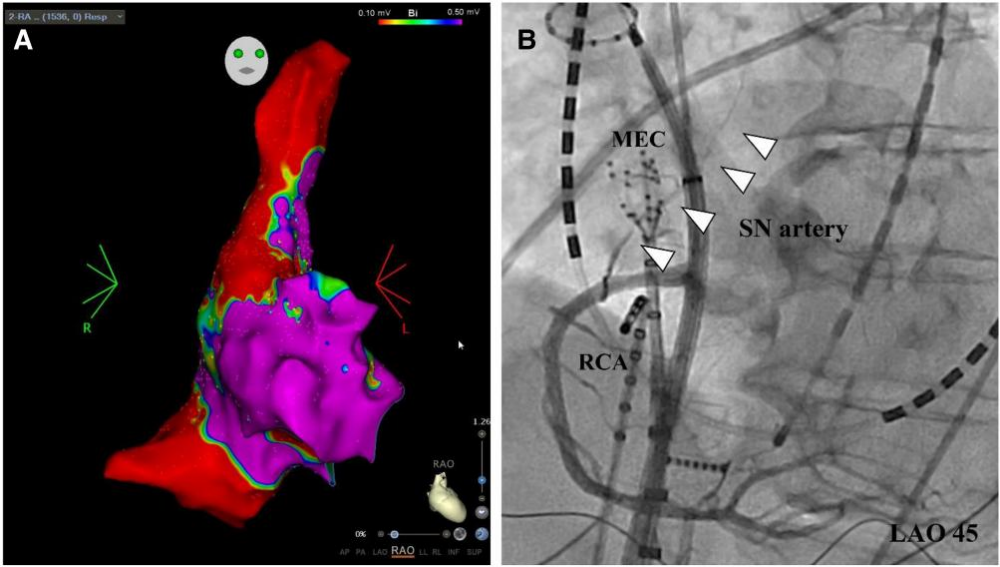
Three-dimensional electroanatomical mapping revealed a local low-voltage area on the high lateral side of the right atrium, which coincided with the sinus node (*A*). (*B*) The multielectrode catheter was located at the low-voltage area of the sinus node, during which coronary angiography was performed. Triangles showed the tracking of the sinus nodeartery that branched from the right coronary artery. The low-voltage area comprised the perfusion area of the sinus node artery.

An 83-year-old female patient with a history of hypertension, dyslipidemia was brought to the emergency department with a chief complaint of general malaise. She had never been diagnosed with arrhythmia but had been experiencing occasional palpitations for a month. On arrival to the hospital, the patient was hypotensive and electrocardiogram (ECG) showed a junctional rhythm with sinus arrest (SA; *Figure 1*). On blood tests, myocardial enzymes were not elevated. Echocardiography revealed mild concentric left ventricular hypertrophy without asynergy. An atrial infarction was suspected because of a sudden onset of SSS; therefore, CAG was performed. A transvenous temporary pacemaker was inserted, and CAG revealed a lone occlusion of the SN artery originating from the proximal right coronary artery (RCA; *Figure 2A*). In left coronary artery, there was no significant stenosis and no branch to perfuse into SN area (*Figure 2D* and *E*). Revascularization for the occluded SN artery was performed in order to recover the SN function. The wire successfully passed through the SN artery using a bougie technique with a microcatheter (*Figure 2B* and *C*). Despite blood flow recovery, SN dysfunction continued. On day 4 after reperfusion, ectopic atrial rhythm appeared, and the temporary pacemaker was removed (*Figure 3A*). A sinus rhythm was finally recovered and stabilized on day 10 (*Figure 3B*). Subsequently, AF became frequent, and the patient complained of palpitations and presyncope. The ECG monitor revealed SA up to 10 s at the time of AF termination, which is known as the bradycardia–tachycardia (BTS). Her CHADS-VASc score was 4, and anticoagulant drug was introduced. Catheter ablation was performed to control AF on day 17. The overdrive suppression test revealed a sinus node recovery time of 6450 ms, suggesting SN dysfunction. Three-dimensional electroanatomical mapping revealed a low-voltage area (LVA) in the high lateral side of the right atrium (RA), which coincided with the SN (*Figure 4A*). No fragmented or late potentials at the LVA could be detected. Coronary angiography revealed that the SN artery perfused the myocardium around the LVA in the higher RA (*Figure 4B*). The atrial infarction was suggested to result in localized LVA around the SN. A multiple ectopy was detected from the right superior pulmonary vein, and they resulted in AF. After pulmonary vein isolation, isoproterenol and atrial rapid pacing did not provoke tachycardia. There was no ectopy from the LVA in the RA. After the CA, she was discharged from the hospital on day 20th. She is free from any symptoms, and no recurrence of AF and SSS for two years. Continued anticoagulant, there is no embolic event with anticoagulant to the present.

## Discussion

This is a rare case of SSS and AF complicated by IAI. We successfully treated them by revascularization and CA. Although the revascularization for SN artery once improved SN function, AF attacks made SN dysfunction apparent as a BTS. Early CA suppressed AF and permanent pacemaker implantation could be avoided.

A few papers have reported IAI caused by acute occlusion of SN artery.^3^ The prevalence of IAI is still unknown, it can be overlooked because it is difficult to diagnose without CAG. As this case, sudden-onset SSS may be the key to suspect IAI. The exact cause and pathogenesis of this disease are not well understood. In this case, the mechanism of IAI was also not clear because the SN artery was too small to insert an imaging catheter such as intravascular ultrasound. The fact that there was no atherosclerotic change in the other coronary arteries supports an embolic infarction. Undiagnosed AF without anticoagulant might result in IAI.

Sinus node artery occlusion is associated with SN dysfunction.^4,5^ Approximately 60% of the SN is supplied by the RCA, 30–40% by the circumflex branch, and 10% by the SN artery originating from both of them.^6,7^ Although the SN is deemed resistant to ischaemia because of the abundance of nutrient vessels,^8,9^ several SSS cases owing to SN artery occlusion during coronary intervention or CA have been reported.^4,5^ These reports indicate that sudden occlusion of major nutrient vessels can cause SN dysfunction. In some reported cases, SN function improved after the revascularization of the occluded SN artery.^4,5^ These reports indicated that SN dysfunction immediately improved after revascularization, whereas others reported that it took a week.^10^ For this reason, we did not proceed with immediate permanent pacemaker implantation, hoping for an improvement in SN function.

Previous reports have been demonstrated that CA is effective for patients with BTS and can avoid pacemaker implantation.^11^ To our knowledge, no studies have reported the electrophysiology of SN dysfunction in patients with IAI. Three-dimensional electroanatomical mapping revealed a scar area in the high RA around the SN, where the SN artery was perfused. This indicates that an atrial infarction can cause LVA in the atrium. Although the LVA may be irreversible, previous reports showed that SN function recovered following reperfusion of the SN artery. This present case also recovered sinus function in spite of the LVA around SN. The possible cause is that reversible oedematous change caused by infarction might result in LVA. However, SN might not be so damaged because voltage map could not express the condition of epicardial side where SN exists. We treated a case of SSS and AF concomitant with IAI. IAI should be considered a differential diagnosis of sudden-onset SSS.

## Data Availability

The authors confirm that written consent for submission and publication of this case report including the images and associated text have been obtained from the patient in line with COPE guidance.

## Lead author biography

I was graduated from Tokushima University and now working as a resident for Hyogo Prefectural Awaji Medical Center in Japan. I am widely interested in clinical medicine of cardiology. The present case impressed me treating a sick sinus syndrome developed with atrial infarction. It is very interesting that there is closed relation between ischaemic heart disease and sinus node dysfunction. I hope this case report may help your practice.

**Consent:** The authors confirm that written consent for submission and publication of this case report including images and associated text has been obtained from the patient in line with COPE guidance.

**Funding:** None declared.
